# *Nocardia cyriacigeorgica* bacteraemia presenting with cytomegalovirus disease and rapidly fatal pneumonia in a renal transplant patient: a case report

**DOI:** 10.1186/1752-1947-5-228

**Published:** 2011-06-23

**Authors:** Simon Namnyak, Mashuk Uddin, Nadia Ahmod

**Affiliations:** 1 Department of Medical Microbiology, Queen's Hospital, Barking, Havering and Redbridge University Hospitals NHS Trust, Romford, RM7 0AG, Essex, UK; 2 Department of Medicine, Queen's Hospital, Barking, Havering and Redbridge University Hospitals NHS Trust, Romford, RM7 0AG, Essex, UK; 3 Molecular Identification Services, Centre for Infections, Health Protection Agency, Colindale, London NW9 5HT, UK

## Abstract

**Introduction:**

*Nocardia cyriacigeorgica *bacteraemia has been described in the setting of profound immunodeficiency in only two previous case reports. In both instances, diagnosis was rapidly facilitated by 16S rRNA gene sequencing of blood culture isolates. To the best of our knowledge, we believe that our case is the first presentation of *N. cyriacigeorgica *bacteraemia associated with acute cytomegalovirus disease in a kidney transplant recipient, which was then followed by severe and fatal pneumonia only seven days later.

**Case presentation:**

We present the case of a 73-year-old Caucasian woman, a renal transplant recipient, with peripheral vascular disease, hypertension, osteoporosis and vascular dementia who was diagnosed with septicemia and pneumonia. In spite of appropriate anti-microbial therapy for nocardial sepsis, she developed severe pneumonia and acute renal failure.

**Conclusion:**

This case illustrates a potential for disseminated nocardial infection to produce clinical syndromes that may be indistinguishable from acute cytomegalovirus disease. An atypical presentation (pneumonia and renal failure) of a rare disease (nocardial septicemia) in the setting of renal transplantation is discussed. This case illustrates that the possibility of severe cytomegalovirus disease should be considered in renal transplanted patients diagnosed with nocardial septicemia who subsequently develop severe sepsis, pneumonia, and renal failure. Molecular diagnosis should readily be available to assist with the prompt diagnosis and treatment of these infections in renal transplant patients.

## Introduction

*Nocardia cyriacigeorgica *bacteraemia has been described in the setting of profound immunodeficiency in only two previous case reports [1,2]. Our case is unusual due to its co-existence with cytomegalovirus (CMV) disease, renal transplantation, and acute rapidity and severity of the subsequent fatal pneumonia. Although there is a link between nocardial septicemia and various medical conditions, its association with acute CMV disease in this setting remains elusive [3,4]. Disseminated nocardiosis and acute CMV disease are known to induce an assemblage of clinical syndromes which are non-specific, requiring rapid, sensitive and specific microbiological techniques to delineate one from the other. In summary, we describe a novel and highly unusual case in which the diagnosis of nocardial septicemia co-existed with acute CMV disease only diagnosed after death.

## Case Presentation

Our patient was a 73-year-old Caucasian woman with a transplanted kidney, peripheral vascular disease, hypertension, osteoporosis and vascular dementia who presented to our hospital with a 10-day history of non-productive cough, vomiting, anorexia, and fever and was non-specifically unwell. The family doctor had recently treated her with trimethoprim for a urinary tract infection. She had received a kidney transplant from her sister 11 years previously due to polycystic kidney disease and she was receiving azathioprine (50 mg once daily), cyclosporine (75 mg twice daily) and prednisolone (7.5 mg once daily).

On examination, our patient was confused, restless and pyrexial with a temperature of 38.4ˌC, respiration rate of 24 breaths/min, blood pressure 111/72 mmHg, and reduced air entry to both lung bases. An echocardiogram showed sinus tachycardia of 129 beats/min. She appeared cachexic and dehydrated. Hematological and biochemical investigations revealed hemoglobin 9.7 g/dl, white cell count 16.0 × 10^9^/L (neutrophils 14.4 × 10^9 ^/L), sodium 136 mmol/L, potassium 5.8 mmol/L, urea 49.7 mmol/L, creatinine 447 μmol/L, bilirubin 18 μmol/L, alanine aminotransferase 60i u/L, alkaline phosphatase 92i u/L, total protein 60 g/L, albumin 25 g/L, and C-reactive protein (CRP) 315 mg/L. A clotted blood sample and ethylene-diamine-tetra-acetic acid (EDTA) whole blood sample for CMV quantitative polymerase chain reaction (PCR) were obtained on the day of death. The clotted sample was positive for CMV immunoglobulin M (IgM) antibodies but negative for CMV immunoglobulin G (IgG) antibodies, and the EDTA blood sample for CMV PCR yielded 11, 899 copies/mL consistent with active CMV disease at the limit of sensitivity of the test at 500 copies/mL.

A chest X-ray revealed a right lower lobe infiltrate and severe kyphoscoliosis. An arterial blood gases analysis on air showed pH 7.424, partial carbon dioxide (pCO_2_) 3.31 kPa, partial oxygen (pO_2_) 32.16 kPa, saturation of peripheral oxygen (SpO_2_) 99.6% and base excess (BE) -6.5 mmol/L. A clinical diagnosis of community-acquired pneumonia was made and given a CURB-65 score of 3/5, giving a prediction of 17% risk of death. Our patient was known to be mildly allergic to penicillin and was therefore started on empirical intravenous antibiotics with imipenem 500 mg every 12 hours to treat possible multiply-resistant Gram-negative bacilli associated with urosepsis, and 1000 mL of sodium chloride intravenous infusion 0.9% was given over eight hours and repeated as appropriate to correct dehydration. Although she remained apyrexial with normal hemodynamic variables for a few more days, her inflammatory markers remained markedly raised and renal function continued to deteriorate gradually despite antibiotics, intravenous fluids and renal support.

On admission to hospital, one set of BacT/ALERT 3D (bioMérieux, Inc., Durham, North Carolina, USA) aerobic and anaerobic blood cultures were collected. After four days of incubation, the aerobic bottle demonstrated long thin Gram positive rod shaped bacterium, identified as *Nocardia *species, sensitive to doxycycline and imipenem, resistant to penicillin, erythromycin, trimethoprim, levofloxacin, clindamycin, rifampicin, teicoplanin and vancomycin based on British Society for Antimicrobial Chemotherapy (BSAC) disk diffusion susceptibility testing. A diagnosis of disseminated nocardiosis was subsequently made and imipenem was continued.

Our patient's clinical condition deteriorated rapidly on day six, developing severe acidotic breathing, reduced level of consciousness (Glasgow Coma Scale 3/15) and acute oliguria, despite adequate fluid resuscitation. Arterial blood gases analysis on 2 L of oxygen showed pH 7.121, pCO_2 _8.41 kPa, pO_2 _8.77 kPa, SpO_2 _85.3.6% and BE -10.1 mmol/L. Hematological and biochemical investigations revealed hemoglobin 6.8 g/dL, white cell count 10.7 × 10^9^/L (neutrophils 9.8.4 × 10^9 ^/L), sodium 136 mmol/L, potassium 5.4 mmol/L, urea 43.5 mmol/L, creatinine 312 μmol/L, bilirubin 6 μmol/L, alkaline phosphatase 83i u/L, total protein 40 g/L, albumin 14 g/L, and CRP 178 mg/L. A chest physician consultation confirmed that our patient was in severe respiratory failure and had severe pneumonia, but was unlikely to benefit from the use of high dose co-trimoxazole and/or valganciclovir respectively to cover for possible *Pneumocystis jirovecii *or CMV infection, and critical care support and assisted ventilation. The patient died on day seven after admission, and her family requested that an autopsy not be performed.

**Figure 1:**
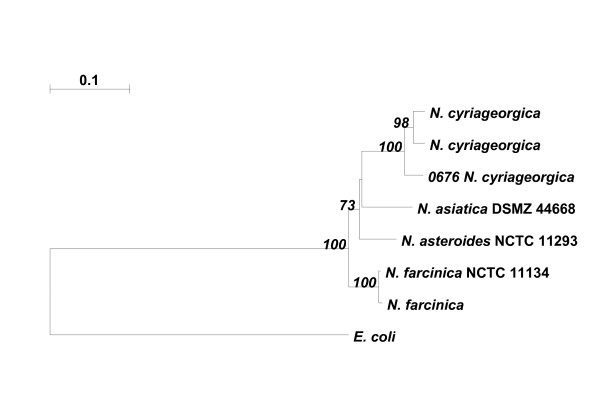
**The tree shows the clustering of *Nocardia *species based on the similarity of their *gyr*B gene sequences**. The scale bar is a measure of the evolutionary distance between species based on the length of the branches. Case Report isolate of *N. cyriageorgica *is number 0676.

The blood culture isolate was confirmed as *N. cyriacigeorgica *at the Molecular Identification Services Unit, Centre for Infections, Health Protection Agency, London, by using partial sequencing of the 16S rDNA and cluster analysis of the gyraseB gene (Figure 1).

## Discussion

The incidence of nocardial infections is believed to be on the rise worldwide as a result of a growing immune-compromised population and improved methods for pathogen isolation and molecular identification [5].

Disseminated nocardiosis is frequently characterized by pulmonary involvement in over 68% of patients, while isolation of the organism, and particularly *N. cyriageorgica*, from blood is very rare [6] even in the presence of severe immune-suppression, cancer and transplantation [2]. These observations have generally remained unexplained in the literature, although it has been suggested that if nocardia infection is clinically suspected, then efforts to optimize the recovery of the micro-organisms in a Bactec blood culture system should be used, with prolonged incubation beyond the usual five days and with frequent and terminal sub-culturing [5]. In the present case, blood cultures incubated in a Bactec blood culture system yielded growth in four days, but unfortunately a sputum sample was not obtained to confirm presence of *Nocardia *in her lung as right lower lobe infiltrate was identified with radiology.

Numerous new species of the *N. asteroides *complex have been recently described, including *N. cyriacigeorgica *[7,8]. This species has been rarely reported and overlooked in human infections so far, since molecular techniques for species identification have not been available [9]. Thus, the present isolate was initially identified only as *Nocardia *species by the API system.

Our patient was treated with intravenous imipenem to which the isolate was sensitive but there was no clinical improvement noted. Until now, no study has established a correlation between the results obtained *in vitro *and the clinical outcome of the patients under treatment with imipenem [10]. Previous studies have suggested clinical response of patients treated with co-trimoxazole was higher than those treated with other antibiotics [6].

In a recent prospective study of 471 consecutive renal transplant recipients, both asymptomatic CMV infection and CMV disease were identified as independent risk factors for overall mortality beyond 100 days post-transplantation [11]. Our patient presented with fever, anorexia, vomiting, coughing, lymphopenia and elevated liver enzymes, symptoms consistent with CMV disease as confirmed by significantly elevated CMV viral load at diagnosis [12]. It is debatable whether our patient could have benefited from early prophylaxis and treatment with ganciclovir if CMV PCR results were known in the early stages of her disease [12].

Although the association of bacteraemic nocardiosis with systemic steroids are described, to the best of our knowledge this is the first case in which nocardiosis is linked to acute CMV disease. There are conflicting reports on whether or not the intensity of immune-suppression in kidney transplant recipients is associated with the risk of CMV infections [11] and therefore further studies are needed to identify any risk factors for CMV infections in kidney transplant recipients to enable prevention strategies for CMV infection to be reconsidered.

## Conclusion

*N. cyriacigeorgica *septicemia and pneumonia in a kidney transplant recipient, co-existing with CMV disease, is rarely reported and may be difficult to diagnose with traditional microbiological methods. New molecular typing schemes are now available to identify new species and assist with the management of patients with invasive disease. Co-existing CMV disease and infections should be diagnosed promptly with CMV PCR to assist with the early treatment and/or prophylaxis with valganciclovir. This case contributes to the spectrum of diseases that may be seen in immune-suppressed patients after kidney transplantation.

## Consent

Written informed consent was obtained from the patient's next-of-kin for publication of this case report and any accompanying images. A copy of the written consent is available for review by the Editor-in-Chief of this journal.

## Competing interests

The authors declare that they have no competing interests.

## Authors' contributions

SN, lead and corresponding author, managed this patient clinically, and helped to draft the manuscript and performed the literature review. MU managed the patient clinically, and helped to draft the manuscript and performed the literature review. NA identified the isolate as *N. cyriacigeorgica *by using partial sequencing of the 16S rDNA and cluster analysis of the gyraseB gene, and helped to draft the manuscript and perform the literature review. All authors read and approved the final manuscript.
